# Insulin Resistance Unveiled: Cushing's Disease in a Patient With Type 1 Diabetes Mellitus and Worsening Glycemic Control

**DOI:** 10.1016/j.aed.2025.12.019

**Published:** 2026-01-05

**Authors:** A. Brooke Baggett, Anna Coppinger, Tiffany Hiatt, Catherine E. Price

**Affiliations:** 1 Wake Forest University School of Medicine Department of Internal Medicine, Division of Endocrinology Medical Center Blvd Winston-Salem, North Carolina; 2 Wake Forest University School of Medicine, Department of Physican Assistant Studies Medical Center Blvd Winston-Salem, North Carolina

**Keywords:** type 1 diabetes mellitus, T1DM, Cushing's disease, insulin resistance, insulin pump, total daily dose, TDD

## Abstract

**Background/Objective:**

Type 1 diabetes mellitus is an autoimmune disease often characterized by endogenous insulin deficiency and often sensitivity to exogenous insulin administration. Cushing's disease, though rare, should be considered as a cause of insulin resistance and increased insulin requirements in individuals with type 1 diabetes mellitus.

**Case Presentation:**

A 21-year-old female with type 1 diabetes mellitus presented with steadily increasing insulin requirements via her hybrid closed-loop insulin pump. She subsequently developed hypertension, weight gain, violaceous striae, and cystic acne. Laboratory evaluation revealed unsuppressed cortisol of 16.6 μg/dL after a 1-mg dexamethasone suppression test, with a simultaneous adrenocorticotropin hormone level of 73.3 pg/mL. Pituitary MRI showed a 1.9 cm sellar mass with local invasion. She underwent transsphenoidal hypophysectomy. Postoperative cortisol was 8.9 μg/dL after intraoperative dexamethasone exposure. Residual hypercortisolism was confirmed, necessitating gamma knife radiation and pharmacologic treatment with a steroidogenesis inhibitor.

**Discussion:**

We present a case of Cushing’s disease due to a corticotropin-secreting pituitary macroadenoma in a young woman with type 1 diabetes. Her initial presentation included rising insulin requirements, followed by overt hypercortisolism. Despite surgery, persistent hypercortisolism required further intervention with gamma knife radiation and osilodrostat. She experienced reductions in both weight and insulin needs, with normalization of cortisol levels on maintenance osilodrostat.

**Conclusion:**

Cushing’s syndrome should be considered in the differential diagnosis of patients with type 1 diabetes and increasing insulin requirements. This case underscores the importance of regular review of automated insulin delivery data and consideration of endocrine causes of insulin resistance and increased insulin requirements in those with type 1 diabetes.

**Highlights**

• Coexistence of hypercortisolism secondary to adrenocorticotropic hormone-producing pituitary adenoma and type 1 diabetes mellitus
• Presentation of Cushing’s disease in individuals with type 1 diabetes mellitus
• Automated insulin delivery utilization in type 1 diabetes with comorbid refractory hypercortisolism

**Clinical Relevance**

This case illustrates the coexistence of type 1 diabetes mellitus and hypercortisolism secondary to corticotropin-secreting pituitary adenoma. It also underscores the importance of regular review of automated insulin delivery doses and inquiring into potential etiologies of increasing insulin requirements when they are noted.

## Introduction

Cushing’s syndrome occurs as the result of prolonged elevation in plasma cortisol which can lead to adverse effects including insulin resistance, hyperglycemia, hypertension, weight gain, immunosuppression, and neurocognitive changes. Cushing’s syndrome can occur due to exogenous exposure to corticosteroids or endogenous cortisol hypersecretion. The most common etiology of endogenous hypercortisolism is Cushing’s disease secondary to a corticotrophin-secreting pituitary tumor. In 90% of cases of Cushing’s disease, patients present with pituitary microadenomas, with only 10% of patients presenting with pituitary tumors >1 cm.^1^

Type 1 diabetes mellitus is an autoimmune condition characterized by T-cell mediated destruction of pancreatic beta cells with ultimate inability to produce insulin and subsequent insulin dependence.^2^ Over the last decade, there has been significant advancement in diabetes management strategies and insulin delivery with creation of hybrid closed-loop insulin pump technology used in conjunction with continuous glucose monitoring systems to provide automated insulin delivery. Within the field of endocrinology, this has required a shift in both the interpretation of glycemic data, insulin utilization data, as well as a pivot to approaching titration of insulin pump settings. Assessment of total daily basal and total daily dose in automated mode is of utmost importance when utilizing automated insulin delivery as the amount of insulin utilized can vary significantly in comparison to fixed quantities seen with use of manual mode in an insulin pump.^2^^,^^3^

Type 1 diabetes mellitus is typically characterized by relative insulin sensitivity, particularly early in the disease course. Patients can develop insulin resistance over time, particularly in the setting of comorbid obesity. However, we present a case of a young woman with type 1 diabetes mellitus presenting with steadily increasing insulin requirements followed by development of overt Cushing's secondary to corticotropin-secreting pituitary macroadenoma. She was utilizing a hybrid-closed loop insulin pump technology with insulin pump download indicating diminished glycemic control despite a steady increase in total daily insulin requirements. This is only the third reported case of Cushing’s disease in a person living with type 1 diabetes mellitus.^4^^,^^5^

## Case Presentation

A 21-year-old female with a history of type 1 diabetes diagnosed at age 11 in the context of admission for diabetic ketoacidosis initially presented to adult endocrinology for routine outpatient diabetes management. Type 1 diabetes mellitus was managed with automated hybrid-closed loop insulin pump technology (Tandem T-slim X:2 with Dexcom G6 continuous glucose monitoring system). Her hemoglobin A1c was 6.2% with a review of her continuous glucose monitoring system indicating time in range of 73% with 21% of blood glucose levels >180 mg/dL. At that time, she reported concerns regarding high insulin requirements despite an active lifestyle as she was running out of insulin for use in pump early. She was noted to have significant prandial insulin requirements with insulin to carbohydrate ratio of 1 unit for every 3.0-4.5 carbohydrates, raising concern for insulin resistance. Over the next 16 months, she had weight gain of 15.8 kg with elevation in blood pressure and worsening hyperglycemia. Review of her insulin pump downloads indicated a steady increase in her total daily insulin requirements of close to 30%, coupled with reduced time in range and increase in HbA1c.

On repeat physical examination, the development of cystic acne, trace pitting pedal edema, and purple violaceous striae on the abdomen, hips, and thighs were observed. She was also noted to have a new elevation of blood pressure to 162/101 mm Hg. She declined exposure to exogenous corticosteroids (including oral, topical or intra-articular formulations). Based on clinical examination and changes in insulin requirements, the decision was made to evaluate for hypercortisolism. Laboratory evaluation at that time revealed unsuppressed 08:00 am cortisol level of 16.6 ug/dL after 1 mg of dexamethasone the evening prior. Dexamethasone level was confirmed to be more than adequate at 418 ng/dL (reference range for 8:00 am level following 1 mg dexamethasone previous evening: 140-295 ng/dL). A simultaneous adrenocorticotropic hormone (ACTH) level was elevated at 73.3 pg/mL (reference range: 7.2-63.3 pg/mL). She was also noted to have midnight salivary cortisol levels of 0.646, 0.290, and 0.350 ug/dL on 3 consecutive evenings (reference range <0.010-0.090 ug/dL).

**Fig. 1:**
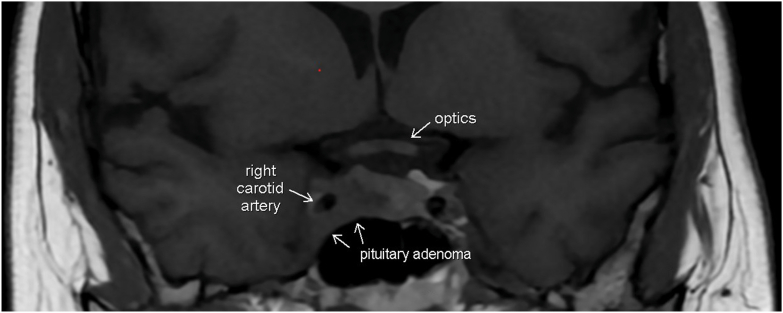
MRI pituitary coronal image revealing sellar mass with invasion of the *right* cavernous sinus; extension around the *right* internal carotid artery.

**Fig. 2:**
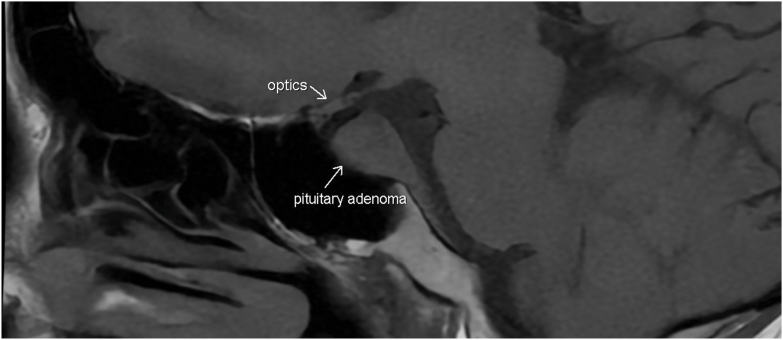
MRI pituitary sagittal image revealing 1.8 × 1.9 cm sellar mass.

She then underwent MRI pituitary with and without gadolinium enhancement which revealed 1.8 × 1.9 cm enhancing sellar mass with invasion of the right cavernous sinus, extension around the right internal carotid artery, as well as posteriorly down the dorsal aspect of the clivus (Figs. 1 and 2). As both hypercortisolism as well as type 1 diabetes mellitus have been implicated as etiologies for lower bone density with subsequent increased risk of osteoporosis later in life, bone densometry was also obtained for this patient. She was found to have low bone mineral density for her age with Z-score of the lumbar spine of −3.2, Z-score of the femoral neck of −2.3, and Z-score of the total hip of −2.8.

She was evaluated by neurosurgery and underwent endoscopic trans-sphenoidal resection of pituitary macroadenoma. Pathology revealed immunoreactivity for neuroendocrine marker INSM1 (Insulinoma-associated protein 1) and adrenocorticotropic hormone. The lesion was negative for immunoreactivity for prolactin, growth hormone, thyroid-stimulating hormone, follicle-stimulating hormone, and beta-luteinizing hormone.

**Fig. 3:**
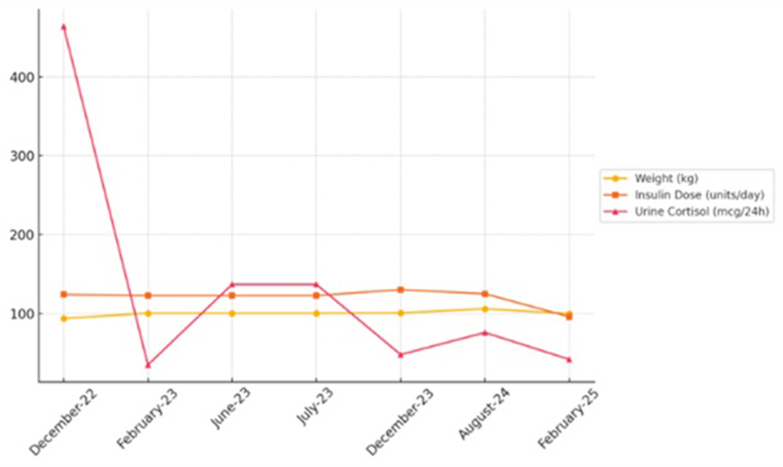
Graphical representation of weight (kg), total daily dose of insulin (units per d), and 24-hour urine cortisol measurements (mcg/24 h).

The cortisol level was 8.9 ug/dL on postoperative day 1. It is notable that she had received 10 mg of intravenous Dexamethasone intraoperatively, raising concern for residual tumor. On postoperative day 3, cortisol level was 4.1 mcg/dL with ACTH level of 94.5 pg/mL. Repeat random cortisol level was 16.3 mcg/dL with simultaneous ACTH level of 63.3 pg/mL. Upon being discharged home, a repeat 24-hour urinary free cortisol was obtained in the outpatient setting and found to be elevated at 464 ug/24 h (reference range: 6-42 ug/24 h), consistent with refractory hypercortisolism (Table, Fig. 3). She was then initiated on osilodrostat, a steroidogenesis inhibitor approved by the FDA in 2020 for use in refractory Cushing’s disease after pituitary surgery. Osilodrostat works via inhibition of 11β-hydroxylase and aldosterone synthase to inhibit the production of cortisol and aldosterone.^6^^,^^7^ She underwent ongoing up titration to a maintenance dose of osilodrostat 7 mg twice daily with additional insulin pump titrations over a 2-year duration. Urinary free cortisol was monitored as this is the gold standard for monitoring refractory Cushing’s and the preferred modality for monitoring cortisol levels in individuals on osilodrostat. Final repeat 24-hour urine free cortisol level normalized to 35 ug/24 h and total daily dose of insulin via automatic insulin delivery system was lower than time of diagnosis of pituitary Cushing’s at 96 units per day despite having had a roughly 27 kg weight gain (Table, Fig. 3).

**Table:** Weight Trends as Well as TDD of Insulin Listed Along With Glycemic Parameters From Automated Insulin Dosing System Tandem T-slim X:2 With Automated Mode Utilizing Decom G6 CGM

Date	Weight (kg)	Total daily insulin dose (units/d)	HbA1c (%)	Time in range (%)	Urine cortisol (mcg/24 h); RR 6-42 mcg/24 h
03/2021	72.7	99.25	6.2	73	
06/2021	74.5	109.56	6.6	73	
12/2021	80.0	117.17	6.9	62	
05/2022	88.5	126.84	6.9	57	
11/2022^a^	-	-	-	-	
12/2022	93.8	124.15	7.0	61	464
01/2023^b^	-	-	-	-	
02/2023					35
06/2023^c^	-	-	-	-	
06/2023	100.4	122.76	7.0	54	
07/2023					137
12/2023	100.9	130.34	6.8	48	48
08/2024	106	125	6.9	58	76
02/2025	100	96	5.8	76	42

Abbreviaton: TDD = total daily dose.

Treatments denoted by asterisk in table include: Twenty-four-h Urine Cortisol Collection Data Included to Highlight Degree of Hypercortisolism.

a Transsphenoidal resection.

b Osilodrostat initiated.

c Gamma knife radiosurgery.

**Fig. 4:**
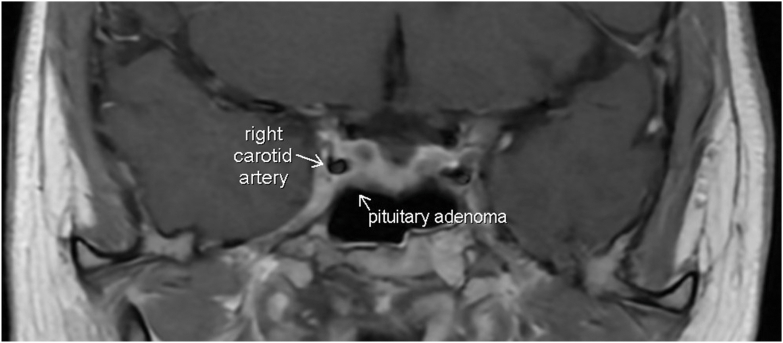
MRI pituitary coronal image revealing *right* eccentric heterogenous enhancing sellar mass which is decreased in size. Redemonstrated residual tissue around the *right* carotid artery.

Due to ongoing hypercortisolism, repeat MRI pituitary with and without gadolinium enhancement was obtained and revealed residual disease in the right sella with right cavernous sinus involvement and extending posteriorly along the dorsal aspect of the clivus (Fig. 4). She had subsequent consultation with neurosurgery at which time the decision made to proceed with single-fraction gamma knife stereotactic radiosurgery. She received additional treatment of gamma knife radiosurgery with dose of 18 Gy to target residual pituitary disease.

## Discussion

We present, to our knowledge, the third reported case of Cushing’s disease due to a corticotropin-secreting pituitary adenoma in an individual with type 1 diabetes. Prolonged hypercortisolism, as seen in this case, is associated with obesity, hypertension, decreased bone density, insulin resistance, and decreased glucose control. Hypercortisolism is most commonly caused by chronic exogenous corticosteroid exposure; however, endogenous hypercortisolemia should be considered as a potential etiology of worsening glycemia and insulin resistance in individuals with diabetes mellitus.

The coexistence of Cushing’s disease secondary to a corticotropin-secreting pituitary macroadenoma in an individual with type 1 diabetes mellitus is exceedingly rare. Furthermore, only 10% of pituitary Cushing’s cases present with macroadenomas at the time of diagnosis. Several studies indicate that smaller lesions at the time of diagnosis and earlier diagnosis of Cushing’s disease are associated with reduced risk of disease recurrence.^6^, ^7^, ^8^ In this case, a young female presented with a macroadenoma at the time of diagnosis and had residual postoperative hypercortisolism requiring gamma knife radiation and pharmacologic intervention with osilodrostat, a steroidogenesis inhibitor approved by the FDA in 2020 for use in refractory Cushing’s disease after pituitary surgery. Osilodrostat works via inhibition of 11β-hydroxylase and aldosterone synthase to inhibit the production of cortisol and aldosterone.^9^^,^^10^ Of the 2 other reported cases of comorbid type 1 diabetes and Cushing’s disease, one individual presented with a macroadenoma at the time of diagnosis. This case occurred in a pediatric male with type 1 diabetes mellitus who was ultimately admitted to the hospital with worsening headaches in the setting of pituitary apoplexy. Prior to hospitalization, this individual showed numerous clinical stigmata of hypercortisolism.

Other contributors to increased insulin resistance, such as obesity, infection, stress, and concurrent glucocorticoids, should also be considered in the differential diagnosis when evaluating etiologies for unexplained changes in glycemic control. However, this case emphasizes the importance of considering the possibility of comorbid Cushing’s disease in persons with type 1 diabetes mellitus. This is imperative to mitigate the consequences of prolonged hypercortisolism and to potentially aid in earlier diagnosis. In this case, declining glucose control and increasing insulin requirements were noted prior to other overt clinical findings of hypercortisolism. Thus, this case also underscores the importance of steadfast evaluation of insulin dose requirements for individuals using continuous insulin infusion devices (particularly hybrid closed-loop automated insulin delivery systems). With growing emphasis on the review and utilization of the one-page ambulatory glucose profile, it is important to also review insulin pump settings and insulin delivery for those utilizing these systems as automated insulin delivery profile for total daily dose can change and should be reviewed at each visit.

## Conclusion

In closing, this case emphasizes the importance of considering secondary endocrine disorders in those living with diabetes mellitus who experience sudden or unexplained changes in glycemic control and insulin requirements. Although rare, coexistence of type 1 diabetes and Cushing’s disease can occur. Prompt recognition and treatment of the underlying Cushing’s disease can lead to significant improvements in insulin sensitivity and glycemic outcomes. This report reinforces the need for multidisciplinary management of vigilant monitoring in patients with coexisting endocrine pathologies, particularly when advanced diabetes technologies are in use. Ultimately, it highlights the critical role of clinical suspicion and timely intervention in optimizing outcomes for complex endocrine cases.

## Disclosure

The authors have no conflicts of interest to disclose

## Author Contributions

All authors made individual contributions to authorship. A.B. and A.C. were involved in the writing and manuscript submission. C.P. contributed in writing and editing. T.H. contributed to the editing, and both T.H. and C.P. were directly involved in the management of this patient. All authors reviewed and approved the final draft.

## Informed patient consent for publication

Patient has provided informed consent for preparation of this case report.
